# HPV testing: a mixed-method approach to understand why women prefer self-collection in a middle-income country

**DOI:** 10.1186/s12889-016-3474-2

**Published:** 2016-08-19

**Authors:** Silvina Arrossi, Silvina Ramos, Cecilia Straw, Laura Thouyaret, Liliana Orellana

**Affiliations:** 1 Centro de Estudios de Estado y Sociedad, Consejo Nacional de Investigaciones Científicas y Técnicas/(CEDES/CONICET), Sánchez de Bustamante 27, C1173AAA Buenos Aires, Argentina; 2 Centro de Estudios de Estado y Sociedad (CEDES), Sánchez de Bustamante 27, C1173AAA Buenos Aires, Argentina; 3 Universidad de Buenos Aires, Facultad de Ciencias Sociales (Buenos Aires, Argentina)/ Centro de Estudios de Estado y Sociedad (CEDES), Sánchez de Bustamante 27, C1173AAA Buenos Aires, Argentina; 4 Instituto Nacional del Cáncer- Ministerio de Salud de la Nación (INC-MSAL), Av. Julio A. Roca 781, C1067ABP Buenos Aires, Argentina; 5 Biostatistics Unit, Faculty of Health, Deakin University, Geelong, 3148 VIC Australia

**Keywords:** Argentina, HPV testing, Self-collection, Acceptability

## Abstract

**Background:**

HPV test self-collection has been shown to reduce barriers to cervical screening and increase uptake. However, little is known about women’s preferences when given the choice between self-collected and clinician-collected tests. This paper aims to describe experiences with HPV self-collection among women in Jujuy, the first Argentinean province to have introduced HPV testing as the primary screening method, provided free of cost in all public health centers.

**Methods:**

Between July and December 2012, data on acceptability of HPV self-collection and several social variables including past screening were collected from 2616 self-collection accepters and 433 non-accepters, and were analyzed using multivariate regression. In addition, in-depth interviews (*n* = 30) and 2 focus groups were carried out and analyzed using thematic analysis.

**Results:**

Quantitative findings indicate that main reasons for choosing self-collection are those reducing barriers related to women’s roles of responsibility for domestic work and work/family organization, and to health care services’ organization. No social variables were significantly associated with acceptability. Among those who preferred clinician-collection, the main reasons were trust in health professionals and fear of hurting themselves. Qualitative findings also showed that self-collection allows women to overcome barriers related to the health system (i.e. long wait times), without sacrificing time devoted to work/domestic responsibilities.

**Conclusions:**

Findings have implications for self-collection recommendations, as they show it is the preferred method when women are given the choice, even if they are not screening non-attenders. Findings also highlight the importance of incorporating women’s needs/preferences in HPV screening recommendations.

**Electronic supplementary material:**

The online version of this article (doi:10.1186/s12889-016-3474-2) contains supplementary material, which is available to authorized users.

## Background

Cervical cancer is one of the primary health problems affecting women. Every year, approximately 500,000 women around the world are diagnosed and around 300,000 die due to the disease, 80 % of them from low-middle income countries [1]. One of the principal determinants of this situation is reduced access to screening services, especially among women in conditions of social vulnerability. The evidence shows that in order to access the Papanicolaou (Pap) test, vulnerable women face social, geographical and cultural barriers as well as obstacles related to the health system’s functioning [2].

The human papillomavirus (HPV) test is a highly effective screening method [3, 4] that offers women the possibility of collecting the sample themselves, with great potential impact in reducing barriers to screening. A number of studies have shown that self-collection is acceptable [5–8], and increases screening uptake [8–11]. In Jujuy, Argentina, the EMA study (Self-collection Modality Trial, initials EMA in Spanish)—a cluster-randomized study to evaluate the effectiveness of HPV test self-collection offered by community health workers (CHWs) during home visits in increasing screening– was carried out [8]. In Jujuy the HPV test has been available free of cost in all public health establishments in the province since 2012 [8]. The women in the study had the possibility of choosing between self-collection at home and collection by a physician at health centers. Understanding the factors associated with women’s choice of self-collection when offered both possibilities is fundamental to incorporating the perspectives of women into the self-collection recommendation, both in terms of potential benefits to women’s health as well as their care preferences. In the framework of the EMA project, a qualitative and quantitative study was carried out with the objective of measuring factors associated with the acceptability of self-collection and understanding women’s reasons for choosing this method. The results are reported in this article.

## Methods

### Setting

The study was nested in a cluster randomized controlled trial of HPV self-collection offered to women by CHWs during home visits (EMA study). The bioethics review committee of Jujuy’s Ministry of Health approved the study. Full characteristics of this study have been extensively described elsewhere [8].

CHWs from the intervention group (self-collection arm) contacted women present at home during their routine visit and instructed them about cervical cancer and HPV testing and offered them the self-collection option. Women who accepted self-sampling were instructed about how to collect the samples.

The study used cross-sectional data collected upon entry into the trial (between July 1 and December 31, 2012) and employed a concurrent mixed methods design, combining quantitative data from all 3049 women that took part in the intervention group and qualitative data from a purposively selected sub-sample of women who accepted self-collection (accepters) and who did not (non-accepters). An informed consent was obtained from all women participating in the study.

### Quantitative component

The quantitative component was based on the analysis of data collected through a survey on acceptability applied by the 94 CHWs from the intervention group on all the women that participated in that group (2616 women who accepted self-collection and 433 women who did not accept self-collection; response rate: 100 %). The questionnaire was applied during the home visit, after the offer of self-collection. CHWs were previously trained for the application of the survey, which was a 7-item closed-ended questionnaire developed by the study team in Spanish (Questionnaire in Additional file 1). Accepters and non-accepters were asked about reasons for their choice; CHWs listened to their answers and then marked all the corresponding options. Information on education level and health insurance was obtained from the Primary Health Care database and through the questionnaire about self-sampling acceptability applied by CHWs. The screening history was extracted from the national information system on screening (SITAM), in which all screening procedures of women who attend public health centers are recorded.

The data from the survey was analyzed using the SPSS 15.0 and SAS 9.3 softwares. Generalized mixed linear models, logit link and binomial distribution with the CHW as a random effect were used to estimate the association between self-collection acceptability and the characteristics of the women (age, health insurance, education level and Pap smears in last 3 years) and the CHWs (gender, urban/rural work setting). Univariate models with each one of these characteristics as the unique fixed effect were fitted. We also ran a multivariate model including variables that were significant in the univariate analysis.

### Qualitative component

The qualitative component centered on in-depth interviews with 30 women and 2 focus groups. We used a convenience sample of women recruited by 10 CHWs selected at random (4 rural and 6 urban); they were asked to recruit 5–8 women for the interviews or focus groups, with variability by age (women in their thirties, forties, fifties, and sixties or over).

Interviews were carried out with both accepters (*n* = 15) and non-accepters (*n* = 15). The interview guide included the following topics: HPV knowledge, reasons for accepting or rejecting the self-collected test, and experience and satisfaction with self-collection. In the case of interviewees that had not accepted the self-collected test, the women were asked about the circumstances surrounding the proposal by the CHW and the possibility of changing their minds in the future to accept self-collection. Interviews were carried out at women’s homes.

Two focus groups with 13 self-collection accepters were carried out. The study included a third group of self-collection non-accepters but it was not possible to recruit those women. The guide included the following topics: knowledge regarding self-collection and reasons for accepting or rejecting the self-collected test.

The qualitative fieldwork was carried out during October and November 2013. Interviews were conducted by trained researchers. Women were consulted by the CHW about their willingness to be interviewed and to participate in the focus groups. Informed consent was obtained.

Data processing and analysis were carried out using Atlas-ti (V.5.0). The verbatim was analyzed thematically and core thematic groupings were identified according to interviewees relevant criteria and were also guided by the study objectives.

The present article reports the results of focus groups and interviews together given data consistency regarding the reasons for accepting or rejecting the self-collected test [12, 13].

## Results

### Characteristics of the population

In total, 3049 eligible women participated in the randomized study and responded to the questionnaire. The distribution of women in the study according to socio-demographic characteristics and those of the CHWs that offered them the self-collection option is summarized in Table 1. As can be seen, 87.9 % of the women were visited by a female CHW and 81.8 % were visited by a CHW in an urban area. Additionally, 42.4 % of the women were aged between 30 and 39 years, 46.3 % had complete primary school/incomplete secondary school, 50.5 % had public health insurance, and 70.8 % had not had a Pap test in the last three years.

**Table 1:** Characteristics of the study population, presented for all women and by self-collection option

	All women	Opted for self-collection		
		Yes	No	*p*-value*
	n(%)	n(%)	n(%)	
Total	3049	2616 (85.8)	433 (14.2)	
Women visited by CHWs of each gender				
Female	2679 (87.9)	2311 (88.3)	368 (85.0)	0.2397
Male	370 (12.1)	305 (11.7)	65 (15.0)	
Area				
Rural	554 (18.2)	439 (16.8)	115 (26.6)	0.2558
Urban	2494 (81.8)	2176 (83.2)	318 (73.4)	
Age (years)				
30–39	1294 (42.4)	1132 (43.3)	162 (37.4)	0.0718
40–49	784 (25.7)	674 (25.8)	110 (25.4)	
50–64	762 (25.0)	651 (24.9)	111 (25.6)	
65+	209 (6.9)	159 (6.1)	50 (11.5)	
Education level				
Tertiary incomplete/complete	367 (14.9)	321 (15.2)	46 (13.0)	0.1641
Secondary complete	468 (19.0)	401 (19.0)	67 (19.0)	
Primary complete/secondary incomplete	1139 (46.3)	992 (47.0)	147 (41.6)	
Never went to school/primary incomplete	488 (19.8)	395 (18.7)	93 (26.3)	
Health insurance				
Private/social security	1326 (49.5)	1113 (48.7)	213 (54.2)	0.0286
Public system	1353 (50.5)	1173 (51.3)	180 (45.8)	
PAP in last three years				
No	2160 (70.8)	1875 (71.7)	285 (65.8)	0.7677
Yes	889 (29.2)	741 (28.3)	148 (34.2)	

*CHWs* community health workers, *Pap* papanicolaou

*Generalized mixed linear model with CHW as a random effect and each factor as a fixed effect

Forty-three women participated in the qualitative study. The ages of the women were: 30–39 years (*n* = 13), 40–49 years (*n* = 12), 50–64 years (*n* = 14) and 65 years or over (*n* = 4). Educational level was the following: never attended school (*n* = 2), incomplete or complete primary education (*n* = 20), incomplete or complete secondary education (*n* = 16), and incomplete or complete tertiary education (*n* = 5). The health insurance distribution was: public system (*n* = 28) and private system or social security (*n* = 15).

### Acceptability according to characteristics of the women and the community health workers

Table 2 shows the analysis of the relationship between acceptability of self-collection and the characteristics of the women and CHWs. In total, 85.8 % (*n* = 2616) of the women who were offered the self-collection option accepted [8]. Neither CHW gender, CHW area nor Pap in the last three years were associated with acceptability. Being older (50 years +) or having a lower education level decreased the likelihood of acceptability, while having public health insurance increased this likelihood. When these last three variables were included in a multivariate model which also included women age, education level and health insurance none of them were statistically significant.

**Table 2:** Association between CHWs and women characteristics and acceptability of self-collection, univariate and multivariate models

	Accepted self-collection	Univariate analysis		Multivariate analysis^a^	
	Yes				
	n(%)	OR (95 % CI)	*p*-value*	OR (95 % CI)	*p*-value*
Total	100				
Women visited by CHW of each gender					
Female	86.26	1			
Male	82.43	0.65 (0.32;1.32)	0.239		
Area					
Rural	79.24	1			
Urban	87.25	1.46 (0.75;2.84)	0.255		
Age (years)					
30–39	87.48	1		1	
40–49	85.97	0.83 (065;1.07)	0.166	0.80 (0.57;1.13)	0.215
50–64	85.43	0.78 (0.63;0.98)	0.032	0.81 (0.56;1.16)	0.263
65+	76.08	0.56 (0.34;0.95)	0.033	0.66 (0.38;1.12)	0.292
Education level					
Tertiary incomplete/complete	87.47	1		1	
Secondary complete	85.68	0.80 (0.50;1.28)	0.365	0.76 (0.48;1.23)	0.271
Primary complete/Secondary incomplete	97.09	0.83 (0.55;1.27)	0.407	0.84 (0.55;1.29)	0.432
Never went to school/primary incomplete	80.94	0.61 (0.38;0.97)	0.039	0.67 (0.40;1.14)	0.141
Health insurance					
Private/social security	83.94	1		1	
Public system	86.70	1.33 (1.03; 1.70)	0.028	1.26 (0.95;1.17)	0.108
PAP in last three years					
No	86.81	1			
Yes	83.35	1.03 (0.81;1.32)	0.767		

*CHW* community health workers, *CI* confidence interval

*Generalized mixed linear model with CHW as a random effect and each factor as a fixed effect

^a^Same generalized linear mixed model including age, education level and health insurance as fixed effects

### Reasons for the acceptance/non-acceptance of HPV test self-collection

Table 3 presents the reasons women mentioned for choosing or not choosing self-collection. Among the 2616 women who chose self-collection, the primary reasons were: gaining time (57.5 %), having other responsibilities that prevented them from attending a health center (47.9 %), and avoiding the process of getting an appointment (44.1 %).

**Table 3:** Reasons for choosing/non-choosing self-collection (multiple answers)

Reasons	%
For choosing self-collection^a^	
Gain time	57.5
Have other responsibilities	47.9
Avoid getting appointments	44.1
Embarrassment at being examined by a health professional	22.7
Large distances to the health center	12.9
Bad experiences with health centers	5.1
Transport problems	4.4
Lack of sample takers or lack of tests	3.2
Other reasons	12.1
For not choosing self-collection^b^	
Trust in the physician	56.6
Fear of hurting herself	38.4
Accustomed to the health centers	20.1
Greater effectiveness of tests at health centers	8.5
Do not have a comfortable place to do it	7.8
Embarrassment	7.3
Other reasons	14.4

^a^Answers provided by 2616 self-collection accepters

^b^Answers provided by 433 self-collection non-accepters

This was also found in qualitative interviews and focus groups, as “Gaining time” by doing self-collection in their homes—without interfering in their family and domestic responsibilities—was the most relevant reason mentioned.

“It makes it easy to do it in your house, you don’t have to leave or go anywhere, and the woman can do it whenever she has time, at night when the kids go to sleep. She dedicates a little bit of time to it and does it herself, I think that’s why lots of women accepted the option of doing it in their homes” (Woman in focus group, 38 years old, rural area).

“The mother is in her house to cook, to look after the children and a ton of other things, she can’t go to the hospital” (Woman interviewed who accepted self-collection, 46 years old, rural area).

“I’m a grandmother, I have to watch my grandchildren, my daughters work […] I can’t make time for myself” (Woman interviewed who accepted self-collection, 58 years old, urban area).

Overcoming barriers to medical care was an important barrier mentioned both in the survey, and interviews and focus groups, especially situations such as appointment shortage, and doctor absenteeism among others.

“It’s true that you save time, and since the community health worker takes care of it and does it in your house you don’t have to go to the doctor, wait for the appointment and all those things, it makes this way much easier” (Woman in focus group, 37 years old, rural area).

“You have to come [to the health center] at three, four [in the morning] for six o’clock when they start giving out appointments […] and sometimes you go and there are no appointments, or you get an appointment at who knows what time in the morning and just your luck, the doctor doesn’t come in, and you’ve been waiting since who knows when” (Woman interviewed who accepted self-collection, 54 years old, urban area).

“There isn’t a [clinical] physician, there’s hardly a pediatrician, or maybe there’s a clinician and no pediatrician, it [the hospital] looks like a first aid clinic at this point” (Woman in focus group, 61 years old, rural area).

Interviews and focus groups revealed that women also accepted the self-collection test given the fact that they perceived this technique as comfortable, easy, fast, painless, voluntary and free, traits that were highly valued. Women also made reference to the respectful way self-collection was offered, the information provided by the CHWs, and -as the background scenario- the trust they have in the CHWs.

“At home everything is comfortable. And they bring it to your house. What else could you ask for? So it’s practically a minute” (Woman in focus group, 62 years old, urban area).

“And in comparison, self-testing doesn’t hurt, in fact one says, "do it this way, it’s easier and doesn’t hurt"” (Woman interviewed who accepted self-collection, 58 years old, urban area).

“I think it has a lot to do with the way they speak to you, how they offer the test, one has to trust the home visitor, right? Because [CHW name] is a woman who always comes around and treats you well” (Woman in focus, 62 years old, urban area).

Among the reasons related to disease prevention, the belief that the test helps to prevent disease, including cancer, was also clearly expressed among the participants in the qualitative study. These references emphasized the possibility of early detection. However, specific references to cervical cancer prevention itself were scarce:

“All of those diseases that have appeared, that it seems like didn’t exist before” (Woman in focus group, 61 years old, rural area).

“I understood it was to detect whether you have something bad that has a solution […] they’re doing this because you’re in time, it’s better to prevent than to cure. I said to myself, “it’s not unnecessary, I don’t lose anything by doing it” (Woman in focus group, 52 years old, urban area).

“What they told me, what little I remember of what they said is that it’s to see if we have HPV, cervical cancer […] so I’m going to do it” (Woman in focus group, 36 years old, urban area).

Lastly, embarrasement-related reasons for the acceptance of self-collection were mentioned by 22 % of women in the survey. In focus groups and interviews, women highlighted the possibility of avoiding the “shame” and also the embarrassment that usually arise with male gynecologists –especially when getting a Pap test–.

“I had a Pap and something was wrong and so they took a second test in the hospital. I was so nervous…there’s always a certain aversion. One has to be willing to open one’s legs in front of a doctor” (Woman in focus group, 42 years old, urban area).

“There are a lot of people out in the country who don’t generally come to the hospital and you say something to a grandmother and she says "I’m not going to be having my intimate parts seen"” (Woman interviewed who accepted self-collection, 47 years old, rural area).

Regarding the role of men in the acceptability of self-collection, references were not consistent. Thus, comments highlighted not only attitudes of rejection or neutral or indifferent reactions but also positive attitudes manifestly in favor of the test. It should be noted that women indicated that conversations with their partners about self-collection had taken place if so, mainly as spontaneous comments but not as requests for permission. This partner issue was not reported in the quantitative survey.

“Some husbands don’t let them. They’re mean […] “Where are you going? I bet you’re going to go see your other men” (Woman interviewed who did not accept self-collection, 63 years old, rural area).

“Some [men] are rigid in that women shouldn’t do it and others no: “it’s ok, you have to take care of yourself, you have to get it done, this that and the other” (Woman interviewed who did not accept self-collection, 66 years old, rural area).

“For example my husband […] he doesn’t say "get it done more frequently" or "no, don’t get it done"” (Woman in focus group, 52 years old, urban area).

“In my case he said, “I’ll help you, I’ll do it for you, explain to me what I have to do and I’ll do it” (Woman interviewed who accepted self-collection, 55 years old, urban area).

Reasons for non-acceptance of self-collection were mainly related to confidence in a trusted physician and the health care system. This was found in results both from quantitative (Table 3) and qualitative methods. In addition, women manifested their insecurity in their ability to correctly use the self-collection test, and their concerns about possibly “hurting” themselves in doing so:

“I trust the physician that sees me […]. They’re used to doing the tests” (Woman interviewed who did not accept self-collection, 49 years old, urban area).

“I don’t know how to do it myself, I’d rather a professional do it. One doesn’t know how far you have to go with that thing” (Women interviewed who did not accept self-collection, 53 years old, rural area).

“I was afraid…maybe I’d scratch myself, hurt myself somewhere, I don’t know. I’ve never put anything inside me, only the doctor when I had to get a test […]. Maybe I’d put it in too far and hurt something inside” (Woman interviewed who did not accept self-collection, 43 years old, urban area).

Other reason for non-acceptance reported by women in both quantitative and qualitative methods was related to the place to perform self-collection. In the survey, 8 % of women mentioned not having a comfortable place to do it. In focus groups and qualitative interviews women mentioned that at home hygiene necessary in a clinical test could not be guaranteed. Therefore, there was a risk that the vaginal sample “could get contaminated and not arrive in good conditions to the laboratory”. For these reasons, they conceived that the result of the test would be more effective if it was taken in a health care facility.

“A house is not hygienic. It’s not a sterilized place for a medical test, the sample could get contaminated with something” (Woman interviewed who did not accept self-collection, 40 years old, urban area).

Some issues arose in qualitative methods that were not reported by women in the survey: first, the concern related to lack of confidentiality in health care facilities. The rationale was that a positive result might be associated with promiscuous sexual behavior of the woman or her male partner, which would in turn cause community disapproval, especially in rural areas.

“Everyone at the hospital is a gossip. When the results come out […] everybody reads them” (Woman interviewed who did not accept self-collection, 33 years old, urban area).

“Also because there’s no…it’s not confidential. Because in the hospital they find something out and they go running to tell one another and so then everyone knows.

INTERVIEWER: Do you think that’s something that happens frequently?

SEVERAL WOMEN: Yes. […]

Lately, yes, […] you don’t really trust going, everywhere you go you’re afraid to tell, to say something because maybe it’ll become known, and they will think negatively about you, and so for that reason you don’t go.[…]

INTERVIEWER: Does that happen in the community?

SEVERAL WOMEN: Yes” (Women in focus group, rural area).

Secondly, cultural and attitudinal concerns related to women’s perception of the health-disease process in general, and to cancer specifically, were also reported as reasons for non-acceptance in interviews and focus groups. In the first case, some women argued that they had a lack of symptoms (pain, inflammation or vaginal discharge). This reason did not seem to be related to self-collection itself, but to their perceptions of the health-disease status defined as the absence or presence of symptoms. To the women interviewed, the absence of symptoms meant there was no disease in their bodies. Consequently, if they believed they were healthy there was no reason for them to get tested for HPV.

“I didn’t feel any pain, I still don’t, I’ve always been healthy like this […]. I thought, it’s clear I don’t have any kind of disorder of the ovaries or anything else” (Woman interviewed that did not accept self-collection, 62 years old, urban area).

“I’m alright, I don’t feel anything. I’m fine,” I said to the health worker, “what for?” (Women interviewed that did not accept self-collection, 66 years old, urban area).

During interviews and focus groups women who accepted self-collection were also asked for what might be the reasons for other women not to accept. Most of them thought that rejection of self-collection was due to lack of interest in their own health, which could even indicate a neglected attitude towards self-care in general.

“Indifference towards their own health […]. And others don’t because they’re lazy, they’re careless women, as we say, they don’t want to go, that’s it, they’re not interested in their bodies” (Woman interviewed who accepted self-collection, 37 years old, urban area).

Among reasons related to beliefs and attitudes regarding cancer, the possibility that screening could result in cancer diagnosis frightened women, thus making them reject the test. Some women expressed that they would rather ignore the situation than face the possibility of being diagnosed with the disease. Given this scenario, waiting for the results was an anxiety-producing factor. Some women also expressed that cancer is a dormant disease that all people have, which can be awaken by introducing a sample-taking device in the vagina or cervix. As a consequence, they rejected screening in general (including self-collection) as a potential cancer inducing factor.

“Fear that the results will say that I’m sick, that I have cancer. I said, “You know, I prefer to die like this, that’s the end of it “[woman laughs] and afterwards, well, I didn’t go” (Woman interviewed who did not accept self-collection, 63 years old, rural area).

“You go around wondering if you are sick, what the results will show, what they’re going to tell you. Fear of knowing the results. They might show that I have cancer” (Woman in focus group, 39 years old, rural area).

“I’m not going to do it…I think that if I let them touch me and get everything done, I don’t know […] if touching it makes it worse” (Woman interviewed who did not accept self-collection, 64 years old, rural area).

## Discussion

In the EMA study, the acceptability of HPV self-collection was high (86 %) and among the women who accepted, almost 100 % were effectively screened [8]. The results of this analysis show that among main reasons for choosing self-collection, were those related to reducing the barriers put up by the health system organization, and those connected to women domestic roles and work responsibilities. These reasons prevailed over cultural or attitudinal reasons such as feelings of shame or embarrassment in gynecological consultation, or their attitude towards screening. The originality of these results resides in the fact that they were produced in a province (Jujuy) where HPV testing is the primary screening test, offered free of cost to all women attending public health centers. Therefore, in our study, women had the opportunity to choose between having the test done in a health care facility and collecting the sample themselves in their homes. Thus, our study did not suffer the bias that can affect acceptability data when the only possibility women have to be screened with HPV-testing, is to perform the self-collection being offered in the research study.

It has been shown that women face multiple barriers to access screening [2]. Geographic accessibility remains a central barrier in most resource-poor settings, as a significant portion of the population at risk for cervical cancer may be located in areas where little or no health care coverage exists [2]. On the other hand, for many women, taking care of their own health takes place in the context of a complex negotiation with other daily responsibilities, such as domestic duties, workplace responsibilities, or taking care of the health of their children and the elderly [14, 15]. These barriers have impact in the context of inefficient health systems with long wait times, shortage of appointments, and disruptions in health care due to different political and institutional problems. In a study on the access to cervical cancer prevention in Argentina, women’s domestic roles appeared as one of the primary barriers to periodic health check-ups [16]. In that study, prioritizing house-keeping tasks and children care were the main arguments justifying lack of time for check-ups. With self-collection women chose a tool that simplifies their health care process and allows them access to screening. Similar results were found in a study carried out in Chile [7].

In countries with organized programs in which women are systematically invited for screening, self-collection has been specifically recommended for screening non-attenders, given the clinical superiority of the HPV test when taken by a medical professional [17, 18]. However, in countries without organized programs screening non-attenders are a more heterogeneous group in terms of the reasons for not getting screened, including those who refuse screening and those would get screened had the services been accessible. On the other hand, there are women who do get screened at great cost in terms of lost workdays and domestic and childcare problems. Therefore, in those settings, self-collection could be offered to all women and allow them to choose based on their preference. Our study shows that even among women who are not under-screened, i.e. who had a Pap in the last three years, HPV test self-collection was the preferred method.

In our study, only a small portion of the women who did not choose self-collection did so because of the greater accuracy of tests taken by medical professionals (9 %), while reasons related to trust in medical care and fear of harming themselves were among the most prevalent. The low importance given to the performance of self-collection is additionally explained by the fact that the screening standard in Argentina is the Pap smear. It was explained to women that they were accessing a test that, although less effective than when carried out by a doctor, was more accurate than cytology [8, 19]. In our study, in which self-collection was carried out in the women’s homes, avoiding embarrassment or shame were relatively less-mentioned reasons. However, in studies in which self-collection was carried out in the context of health facilities [20–22], embarrasement-related barriers had greater weight. In two studies carried out in Mexico and the United States [20, 21], such reasons were mentioned by 38 % and 56 % of the women surveyed, respectively. In these studies the women were health system users and the question regarding their preference for self-collection was asked in the context of a gynecological exam with a Pap smear taken by a health professional. Similar results were found in a study carried out in El Salvador [22], where the most cited reason for preferring self-collection was greater privacy or less embarrassment (30 %), whereas practical reasons such as time convenience was cited by only 8.5 % of them. In that study, women were screened at health centers, and reasons for acceptability were also surveyed following self-collection performed in a private room, after HPV testing sampled by a medical doctor. It is likely that given the immediate comparison between self-collection (in a space that assures the privacy and intimacy of the women) and a test taken by a doctor, barriers related to embarrassment take on a more important role. On the other hand, it is important to highlight that in these studies, the women screened were those who responded to an invitation to be screened at health centers, and therefore, practical and geographical barriers to screening might be less important for this specific group.

Other cultural and attitudinal motivations for not choosing HPV test self-collection, mentioned both in our study as well as others [7, 23] was fear that the HPV test result would show cancer or that screening could awaken dormant cancer. These reasons are not specifically related to self-collection but rather to the fear associated with cancer diagnosis and/or lay beliefs about the disease, its causes and prevention. A study carried out in Argentina with healthy women and men on social images of different types of cancer (uterine, breast and colorectal cancer) found that people had an unitary conceptualization of cancer, that is, they conceived of the different types of cancer as a single, latent and dormant disease, that for different reasons sometimes manifested itself (“awakened”) in different parts of the body [24]. Study authors proposed that these ideas about cancer jeopardize the concept of prevention, as the awakening of the disease is understood as a fatalistic and unpredictable fact. Fear of cancer diagnoses as a cause for not seeking screening has been reported in studies looking into barriers in cervical cancer prevention using other screening methods (Pap, visual inspection) [16, 25, 26] and breast cancer [27, 28].

In our study, partner attitudes towards screening were not mentioned as a factor limiting the possibility of carrying out a self-collected test. This is an important finding given that partner support has been mentioned as a key factor for screening in numerous studies [2]. Among women surveyed in Mexico [23], 66 % stated that male opposition could be an obstacle to carrying out self-collection. The privacy context in which collection occurred and the participation of CHWs, representatives of the health system, could have been factors that reduced partner opposition. However, it is also possible that this influence could vary with the cultural context in which the strategy is applied. Another study, also carried out in Mexico (Morelos) [20], found that partner preference was not a main reason to choose self-collection. Partner support is dependent on cultural beliefs and norms about sexual health, gynecological care, and gender inequality. Therefore, in each specific context, the way HPV-testing and self-collection is explained can make a significant difference in partner acceptance. More context-specific evidence about this subject is needed.

Our study also showed the importance of reasons for preferring self-collection related to the hospital administrative staff and CHWs, which has not been described in previous studies. In effect, women mentioned that the form and content of the messages transmitted by CHWs and the prior trust they had in them favored acceptance of self-collection. In Jujuy, where the EMA project was carried out, CHWs have a long tradition in the health system, are respected for their knowledge of local and contextual particularities, and have a good connection with the majority of the population, connection that has been developed over years of work [29]. In a study carried out among Haitian women in the United States [30], trust in CHWs was mentioned by authors as a likely key factor in the positive response to HPV test self-collection. In that study, the analysis highlighted the Haitian ancestry common to the immigrant women studied and the health workers that participated in the study.

In contrast, fear of non-confidential treatment of test results emerged as a reason for not choosing self-collection of the HPV test. This distrust was expressed in relation to the administrative staff of health facilities, and regarding difficulties with CHWs delivering the results. In this way, the possible stigma that could follow the disclosure of HPV-positive result, especially in rural areas, was mentioned. In Argentina, this relationship between sexual transmission and social reproach is not new. A study found that, upon being questioned regarding the idea that sexually transmitted diseases were spread by women, men generally agreed with the statement while only some women agreed. Similarly, men identified women who spread sexually transmitted diseases as “street women,” “women of the night,” and “dirty women” [31].

HPV test self-collection does not relieve women with positive test results from undergoing triage and diagnostic tests, and later treatment if necessary. Compliance with further follow-up among self-sample HPV-positive women varied in trials between 41 % and 100 % [17]. For this reason, in the framework of self-collection application, special care should be taken in creating the referral and counter-referral network and in implementing strategies that reduce barriers to access to the second and third level of care. If barriers to access persist among HPV positive women who need follow-up and treatment, then the self-collection strategy will be ineffective in reducing cervical cancer incidence and mortality.

This study has some limitations that deserve mention. We were not able to carry out a focus group with women who did not accept self-collection. Therefore, we are not reporting issues that might have arisen in the focus group and have not in the personal interviews. Another limitation is the time elapsed between the self-collection offer and the qualitative study, with potential recall bias. However, consistency found between results from the quantitative survey (carried out at the moment of the offer of self-collection) and results from the qualitative study suggest that recall bias was not an issue in this study. Finally, participating women were recruited by CHWs and may have been more willing to participate based on their relationship with them.

Our study provides evidence about reasons for accepting or non-accepting self-collection when offered by CHWs that are part of the Jujuy primary health care system and therefore a question remains about generalizability of these results. However, CHWs are involved in many countries in the offer of a wide range of health services, therefore, incorporating HPV-self collection into CHWs work could be feasible. In those settings, results from our study will be very relevant and useful for informed design of strategies to offer self-collection.

## Conclusions

Women highly accepted the incorporation of this new screening strategy, not dependent on the intervention of a medical professional. This study shows that reasons expressed for performing self-collection are above all related to their work/domestic role, and to the complexity of organization of the health system, which in general does not take into account the preferences and needs of women. Given that women have responsibility for domestic labor, child-rearing and education, and for care of the sick and the elderly, they tend to defer care of their own health. Self-collection gives them the possibility to prevent cervical cancer with a highly effective strategy, especially when compared to the Pap test, and at the same time allows for greater autonomy in caring for their health.

## Abbreviations

CHWs, community health workers; EMA, self-collection modality trial, initials EMA in Spanish); HPV, human papillomavirus; Pap, papanicolaou

## Additional file

Additional file 1: Questionnaire on acceptability of HPV self-collection. (DOCX 282 kb)

## References

[1] Ferlay J, Soerjomataram I, Ervik M, Dikshit R, Eser S, Mathers C, et al. GLOBOCAN 2012 v1.0, cancer incidence and mortality worldwide: IARC CancerBase No.11. IARC-WHO. 2013. http://globocan.iarc.fr. Accessed 13 Nov 2015.

[2] Bingham A, Bishop A, Coffey P, Winkler J, Bradley J, Dzuba I (2003). Factors affecting utilization of cervical cancer prevention services in low-resource settings. Salud Publica Mex.

[3] Sankaranarayanan R, Nene B, Shastri S, Jayant K, Muwonge R, Budukh AM, et al. HPV screening for cervical cancer in rural India. N Engl J Med. 2009;doi: 10.1056/NEJMoa0808516 10.1056/NEJMoa0808516 19339719

[4] Ronco G, Giorgi-Rossi P, Carozzi F, Confortini M, Dalla Palma P, Del Mistro A (2010). Efficacy of human papillomavirus testing for the detection of invasive cervical cancers and cervical intraepithelial neoplasia: a randomised controlled trial. Lancet Oncol.

[5] Giorgi Rossi P, Marsili LM, Camilloni L, Iossa A, Lattanzi A, Sani C, et al. The effect of self-sampled HPV testing on participation to cervical cancer screening in Italy: a randomised controlled trial (ISRCTN96071600). Br J Cancer. 2011;doi: 10.1038/sj.bjc.6606040 10.1038/sj.bjc.6606040 PMC3031894 21179038

[6] Zehbe I, Moeller H, Severini A, Weaver B, Escott N, Bell C, et al. Feasibility of self-sampling and human papillomavirus testing for cervical cancer screening in First Nation women from Northwest Ontario, Canada: a pilot study. BMJ Open. 2011;doi: 10.1136/bmjopen-2010-000030 10.1136/bmjopen-2010-000030 PMC3191400 22021733

[7] Léniz J, Barriga MI, Lagos M, Ibáñez C, Puschel K, Ferreccio C (2013). HPV vaginal self-sampling among women non-adherent to Papanicolaou screening in Chile. Salud Publica Mex.

[8] Arrossi S, Thouyaret L, Herrero R, Campanera A, Magdaleno A, Cuberli M, et al. Effect of self-collection of HPV DNA offered by community health workers at home visits on uptake of screening for cervical cancer (the EMA study): a population-based cluster-randomised trial. Lancet Glob Health. 2015;doi: 10.1016/S2214-109X(14)70354-7 10.1016/S2214-109X(14)70354-7 25617202

[9] Szarewski A, Cadman L, Mesher D, Austin J, Ashdown-Barr L, Edwards R, et al. HPV self-sampling as an alternative strategy in non-attenders for cervical screening – a randomised controlled trial. Br J Cancer. 2011; doi: 10.1038/bjc.2011.48 10.1038/bjc.2011.48 PMC3065284 21343937

[10] Virtanen A, Nieminen P, Luostarinen T, Anttila A. Self-sample HPV tests as an intervention for nonattendees of cervical cancer screening in Finland: a randomized trial. Cancer Epidemiol Biomarkers Prev. 2011; doi: 10.1158/1055-9965.EPI-11-0307 10.1158/1055-9965.EPI-11-0307 21752985

[11] Verdoodt F, Jentschke M, Hillemanns P, Racey CS, Snijders PJ, Arbyn M. Reaching women who do not participate in the regular cervical cancer screening programme by offering self-sampling kits: a systematic review and meta-analysis of randomised trials. Eur J Cancer. 2015;doi: 10.1016/j.ejca.2015.07.006 10.1016/j.ejca.2015.07.006 26296294

[12] Kitzinger J (1995). Qualitative research. Introducing focus groups. BMJ.

[13] Liamputtong P (2011). Focus Group Methodology: Principles and Practice.

[14] Cadman L, Ashdown-Barr L, Waller J, Szarewski A. Attitudes towards cytology and human papillomavirus self-sample collection for cervical screening among Hindu women in London, UK: a mixed methods study. J Fam Plann Reprod Health Care. 2015;doi: 10.1136/jfprhc-2013-100705 10.1136/jfprhc-2013-100705 PMC4514978 24521934

[15] Goldman MB, Troisi R, Rexrode KM (2013). Women and health.

[16] Zamberlin N, Thouyaret L, Arrossi S (2013). Lo que piensan las mujeres: conocimiento y percepciones sobre cáncer de cuello de útero y realización del Pap.

[17] Arbyn M, Verdoodt F, Snijders PJ, Verhoef VM, Suonio E, Dillner L, et al. Accuracy of human papillomavirus testing on self-collected versus clinician-collected samples: a meta-analysis. Lancet Oncol. 2014;doi: 10.1016/S1470-2045(13)70570-9 10.1016/S1470-2045(13)70570-9 24433684

[18] Rozemeijer K, de Kok IM, Naber SK, van Kemenade FJ, Penning C, van Rosmalen J, et al. Offering self-sampling to non-attendees of organized primary HPV screening: when do harms outweigh the benefits? Cancer Epidemiol Biomarkers Prev. 2015;doi: 10.1158/1055-9965.EPI-14-0998 10.1158/1055-9965.EPI-14-0998 25432954

[19] Lazcano-Ponce E, Lorincz AT, Cruz-Valdez A, Salmerón J, Uribe P, Velasco-Mondragón E, et al. Self-collection of vaginal specimens for human papillomavirus testing in cervical cancer prevention (MARCH): a community-based randomised controlled trial. Lancet. 2011;doi: 10.1016/S0140-6736(11)61522-5 10.1016/S0140-6736(11)61522-5 22051739

[20] Dzuba IG, Díaz EY, Allen B, Leonard YF, Lazcano Ponce EC, Shah KV (2002). The acceptability of self-collected samples for HPV testing vs. the Pap test as alternatives in cervical cancer screening. J Womens Health Gend Based Med.

[21] Anhang R, Nelson JA, Telerant R, Chiasson MA, Wright TC (2005). Acceptability of self-collection of specimens for HPV DNA testing in an urban population. J Womens Health (Larchmt).

[22] Rosenbaum AJ, Gage JC, Alfaro KM, Ditzian LR, Maza M, Scarinci IC, et al. Acceptability of self-collected versus provider-collected sampling for HPV DNA testing among women in rural El Salvador. Int J Gynecol Obstet. 2014;doi: 10.1016/j.ijgo.2014.02.026 10.1016/j.ijgo.2014.02.026 24880188

[23] Arriba LN, Enerson CL, Belinson S, Novick L, Belinson J. Mexican cervical cancer screening study II: acceptability of human papillomavirus self-sampler. Int J Gynecol Cancer. 2010;doi: 10.1111/IGC.0b013e3181f58678 10.1111/IGC.0b013e3181f58678 21051987

[24] Instituto Nacional del Cáncer (2013). Imágenes sociales del cáncer: estudio nacional para orientar la comunicación social.

[25] Lazcano-Ponce EC, Castro R, Allen B, Nájera P, de Ruíz PA A, Hernández-Avila M (1999). Barriers to early detection of cervical-uterine cancer in Mexico. J Womens Health.

[26] Ndikom CM, Ofi BA. Awareness, perception and factors affecting utilization of cervical cancer screening services among women in Ibadan, Nigeria: a qualitative study. Reprod Health. 2012;doi: 10.1186/1742-4755-9-11 10.1186/1742-4755-9-11 PMC3539913 22866676

[27] Jones CE, Maben J, Jack RH, Davies EA, Forbes LJ, Lucas G, et al. A systematic review of barriers to early presentation and diagnosis with breast cancer among black women. BMJ Open. 2014;doi: 10.1136/bmjopen-2013-004076 10.1136/bmjopen-2013-004076 PMC3927711 24523424

[28] Jones CE, Maben J, Lucas G, Davies EA, Jack RH, Ream E. Barriers to early diagnosis of symptomatic breast cancer: a qualitative study of Black African, Black Caribbean and White British women living in the UK. BMJ Open. 2015;doi: 10.1136/bmjopen-2014-006944 10.1136/bmjopen-2014-006944 PMC4360845 25770231

[29] Drovetta RI, Drovetta RI, Rodríguez ML (2010). Prestadores de servicio de salud alopática y usuarios indígenas en la Puna de Atacama. Padecimientos en grupos vulnerables del interior de Argentina: procesos históricos y actuales de salud, enfermedad y atención.

[30] Barbee L, Kobetz E, Menard J, Cook N, Blanco J, Barton B, et al. Assessing the acceptability of self-sampling for HPV among Haitian immigrant women: CBPR in action. Cancer Causes Control. 2010;doi: 10.1007/s10552-009-9474-0 10.1007/s10552-009-9474-0 19943103

[31] Gogna M, Ramos S, Parker R, Barbosa MR, Aggleton P (2000). Gender stereotypes and power relations: unacknoledged risks for STDs in Argentina. Framing Sexual Subject.

