# Supplementary material for: HPV testing: a mixed-method approach to understand why women prefer self-collection in a middle-income country
Source: BMC Public Health. 2016 Aug 19;16:832. doi: 10.1186/s12889-016-3474-2 (PMC4990977; doi:10.1186/s12889-016-3474-2)
Supplement: Additional file 1: — Questionnaire on acceptability of HPV self-collection. (DOCX 282 kb) [file 12889_2016_3474_MOESM1_ESM.docx]

**EMA PROJECT: QUESTIONNAIRE**

**SELF-COLLECTION ARM**

**A**

| **Date:**___________________________**CHW name and surmane:**______________________________________  **Woman name and surname:**________________________**Unique national identifier number:**_____________  **Birthday date:** ________________ **Address:** _____________________________________________________ |
| --- |

**B**

| Already HPV-tested this year | YES ˽ | NO ˽ |
| --- | --- | --- |

**C**

| Pregnant | YES ˽ | NO ˽ |
| --- | --- | --- |
| Had hysterectomy | YES ˽ | NO ˽ |
| Under treatment for  precancerous lesions or cervical cancer | YES ˽ | NO ˽ |
| A disability does not allow the woman to perform self-collection  (do not ask, complete after observation) | YES ˽ | NO ˽ |

***IF ANY OF THESE ANSWERS IS YES, END OF SURVEY.***

**D**

| Informed consent signed | YES ˽ | NO ˽ |
| --- | --- | --- |

***IF THE ANSWER IS NO, END OF SURVEY.***

**E**

| She chose self-collection | YES ˽ | NO ˽ |
| --- | --- | --- |
| If she chose self-collection | SAMPLE DELIVERED INMEDIATELY ˽ | SAMPLE DELIVERED THE DAY/DAYS AFTER ˽ |

**F**

| ***FOR WOMEN CHOOSING SELF-COLLECTION***  **ASK: Why did you choose self-collection? Listen to the answer and mark all the corresponding options.** |  |
| --- | --- |
| Embarrassment at being screened by a health professional: | ˽ |
| Bad experiences with health centers: | ˽ |
| Have other responsibilities (children, job, home, etc): | ˽ |
| Large distances to the health center: | ˽ |
| Transport problems: | ˽ |
| Gain time: | ˽ |
| Avoid getting appointments: | ˽ |
| Other reason: | ˽ |
| Please specify:  ----------------------------------------------------------------------------------------------------- | |

| ***FOR WOMEN NOT CHOOSING SELF-COLLECTION***  **ASK: Why did you choose to be screened at the health center? Listen to the answer and mark all the corresponding options.** |  |
| --- | --- |
| Fear of hurting herself: | ˽ |
| Embarrassment: | ˽ |
| Accustomed to attend the health center: | ˽ |
| Do not have a comfortable place to do it: | ˽ |
| Trust in the physician: | ˽ |
| Mobile units available: | ˽ |
| Greater effectiveness of test at health centers: | ˽ |
| Other reason: | ˽ |
| Please specify:  ------------------------------------------------------------------------------------------------------ | |

**G**

| GENERAL OBSERVATIONS: __________________________________________________________________________________________________________________________________________________________________________________________________________________________________________________________________________________________________________________________________________________­­­­­­­­­­­­­­­­­­­­______________ |
| --- |

**ANNEX**

**Ask to the woman:**

**¿Which is your highest education level attained? (select the apropriate option)**

| **Never went to school** |  |
| --- | --- |
| **Primary incomplete** |  |
| **Primary complete** |  |
| **Secondary incomplete** |  |
| **Secondary complete** |  |
| **Tertiary incomplete** |  |
| **Tertiary complete** |  |
| **Universitary incomplete** |  |
| **Universitary complete** |  |

**¿Which is your Health insurance system? (select the corresponding option)**

| **Private** |  |
| --- | --- |
| **Social security** |  |
| **Public system** |  |
